# Performance of Acoustic, Electro-Acoustic and Optical Sensors in Precise Waveform Analysis of a Plucked and Struck Guitar String

**DOI:** 10.3390/s25216514

**Published:** 2025-10-22

**Authors:** Jan Jasiński, Marek Pluta, Roman Trojanowski, Julia Grygiel, Jerzy Wiciak

**Affiliations:** AGH University of Krakow, Department of Mechanics and Vibroacoustics, Av. Mickiewicza 30, 30-059 Krakow, Poland; pluta@agh.edu.pl (M.P.); roman.cz.trojanowski@agh.edu.pl (R.T.); wiciak@agh.edu.pl (J.W.)

**Keywords:** non-contact sensing, sensor comparison, laser Doppler vibrometer (LDV), electro-acoustic transducer, musical acoustics, string vibration, electric guitar, waveform analysis

## Abstract

This study presents a comparative performance analysis of three sensor technologies—microphone, magnetic pickup, and laser Doppler vibrometer—for capturing string vibration under varied excitation conditions: striking, plectrum plucking, and wire plucking. Two different magnetic pickups are included in the comparison. Measurements were taken at multiple excitation levels on a simplified electric guitar mounted on a stable platform with repeatable excitation mechanisms. The analysis focuses on each sensor’s capacity to resolve fine-scale waveform features during the initial attack while also taking into account its capability to measure general changes in instrument dynamics and timbre. We evaluate their ability to distinguish vibro-acoustic phenomena resulting from changes in excitation method and strength as well as measurement location. Our findings highlight the significant influence of sensor choice on observable string vibration. While the microphone captures the overall radiated sound, it lacks the required spatial selectivity and offers poor SNR performance 34 dB lower then other methods. Magnetic pickups enable precise string-specific measurements, offering a compelling balance of accuracy and cost-effectiveness. Results show that their low-pass frequency characteristic limits temporal fidelity and must be accounted for when analysing general sound timbre. Laser Doppler vibrometers provide superior micro-temporal fidelity, which can have critical implications for physical modeling, instrument design, and advanced audio signal processing, but have severe practical limitations. Critically, we demonstrate that the required optical target, even when weighing as little as 0.1% of the string’s mass, alters the string’s vibratory characteristics by influencing RMS energy and spectral content.

## 1. Introduction

The initial transient of a vibrating string contains critical information about the energy input, excitation mechanism, and interaction with the instrument body. In stringed instruments such as the guitar, this short-lived phase is perceptually important for timbre and identity recognition, and it plays a key role in both musical expression and sound synthesis applications. Despite its significance, the initial transient is difficult to capture with precision. It exhibits high-frequency content, non-linear behavior, and rapid temporal evolution, requiring measurement techniques with excellent time and spatial resolution. Furthermore, the mechanical coupling between components complicates the isolation of the string’s contribution using conventional acoustic recording methods.

Previous research has utilized various sensors to study instrument string vibrations, including microphones [1,2], electromagnetic pickups [2,3,4], contact pickups [5], and more recently, optical systems such as laser Doppler vibrometers (LDVs) [1,6], position sensitive detectors (PSD) [7], and high-speed cameras [1,8,9]. However, comparative studies evaluating the strengths and limitations of these methods—particularly under controlled excitation and measurement conditions—remain limited. Such studies have been conducted for instrument plate and body vibration measurements [10,11], but not for strings. Their results cannot be directly transferred, as strings differ substantially in mass, dimensions, and vibration amplitude. This gap hinders the ability to select suitable sensors for specific applications, such as instrument design [12], modeling of instruments [13], strings [14,15] and pickups [16], sound synthesis [17], and performance analysis [18].

This study provides a comparative evaluation of three sensor technologies—laser Doppler vibrometer, microphone, and magnetic pickup—for measuring guitar string vibration under varied excitation conditions. The laser Doppler vibrometer (LDV) provides high-resolution, non-contact measurement of the string velocity by detecting the Doppler shift of reflected laser light. This makes it particularly well suited for detailed analysis of vibrational modes and transient behaviour. The electromagnetic pickup, integral to the functioning of electric guitars, measures string motion through variations in magnetic flux induced by the vibration of ferromagnetic strings near a magnet and coil. While this signal reflects the transverse velocity of the string at the pickup location, it is inherently shaped by the pickup’s position, design, and magnetic characteristics. The microphone detects acoustic pressure variations in air generated by the vibrating string. Although not commonly used with electric guitars, the focus on the very starting moments of the string’s vibration means that we are not interested in the amplification that would come from the vibration of a guitar body in an acoustic guitar. Additionally, the microphone captures the radiated sound field, which is directly related to perceived timbre. Therefore, it is worth investigating whether precise string movement data can be extracted from such recordings. Together, these methods provide complementary perspectives on string vibration, ranging from precise physical characterization to musically functional signal capture. Measurements were conducted using a specially constructed stand, a simplified electric guitar platform, and three repeatable excitation methods. By analyzing the recordings, we assess each sensor’s effectiveness in capturing selected vibro-acoustic phenomena related to excitation method and measurement position. The findings offer practical guidance for sensor selection in experimental design.

## 2. Experimental Method

### 2.1. Experimental Setup

**Figure 1:**
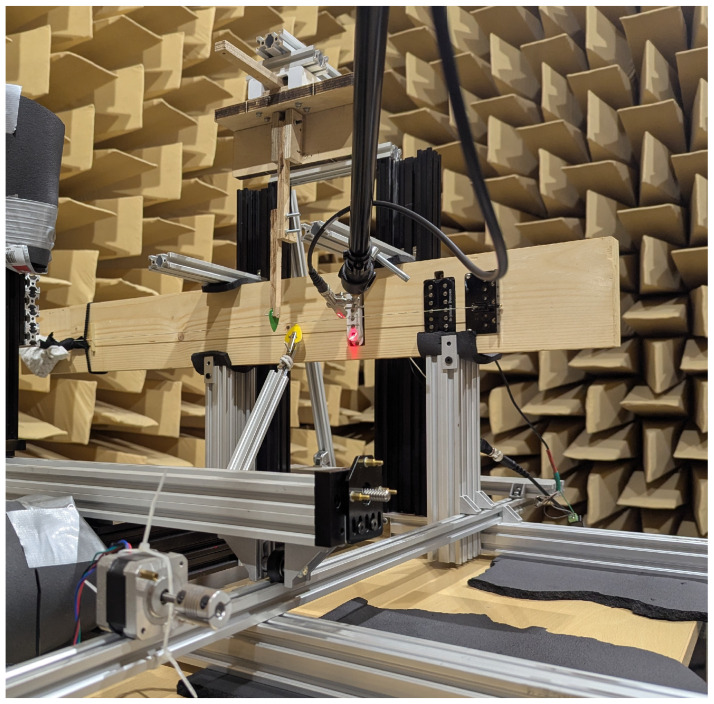
Simplified electric guitar model mounted in the experimental stand.

The objective of the experimental setup was to eliminate as many external variables from the measured string vibrations as possible. To achieve this, a specialized stand was utilized to provide a stable mounting platform for the setup. A heavily simplified electric guitar served as the mounting for the pickups and string. It retains the proportions of a standard instrument, incorporating a guitar bridge, nut, key, and pickups, while featuring a simplified shape. This design ensures that the string vibrations are representative of a standard guitar while allowing for more repeatable mounting. The exact dimensions and design considerations that went into the creation of this simplified model are described in [4]. The corpus is clamped into the stand using dense foam, which provides stable placement while limiting vibration transfer between the instrument and the stand. This setup is shown in Figure 1. All measurements were conducted in a large anechoic chamber in the Department of Mechanics and Vibroacoustics of the AGH University of Krakow to minimize the impact of noise and interference on the experimental results.

The distance between the guitar bridge and the nut was 645 mm. The guitar pickups were mounted to the instrument and so the measuring point was chosen above them at 42 mm and 157 mm from the guitar bridge. To better observe certain vibro-acoustic phenomena caused by different excitation methods, the measuring point was selected on the shorter segment of the string, as divided by the excitation point. Consequently, the excitation point was chosen at 240 mm from the bridge to avoid proximity to any nodes of the first ten harmonic frequencies (excitation point to string length ratio equal to 0.372).

Experiments were conducted using two string diameters: a 1.17 mm (0.046 inch) steel core wound string and a 0.28 mm (0.011 inch) unwound steel string. D’Addario XL Nicklewound strings (D’Addario & Co., Farmingdale, NY, USA) were used. Strings were tuned to E2 (82.41 Hz) and B3 (246.94 Hz), respectively.

### 2.2. Measurement Methods

Three separate methods were utilized to measure the vibration of the string. To allow for the highest level of comparability between achieved results, the aim is for them to measure the vibration of the string at the same time, at the same point, and in the same direction.

Firstly, a Polytec VibraGo single-point laser Doppler vibrometer (LDV) was used (Polytec GmbH, Waldbronn, Germany). The vibrometer was positioned approx. 2 m away from the string and oriented perpendicularly to it. The vibrometers autofocus function was used to focus the laser onto the string. Due to the minimal size of the point at which vibrations are measured, it was not possible to conduct measurements without the use of a reflective sticker placed on the string. Without this sticker, the string would move outside the laser beam, resulting in sudden jumps in the recorded signal and incorrect measurements. A 3 × 4 mm rectangle of reflective tape, weighing 0.0056 g, proved sufficient to address this issue. This sticker and its influence on the vibration of the string will be analyzed and discussed in detail in Section 5.

The second measurement method involved a GRAS G46AE measurement microphone positioned near the string (GRAS Sound & Vibration, Holte, Denmark). The placement was as close as possible to the selected measurement point; however, due to the path of the laser vibrometer, the microphone had to be elevated. Consequently, the measurement direction is not identical.

The final measurement method utilized electromagnetic pickups mounted in the simplified electric guitar. Two pickups were used as follows: a single-coil Seymour Duncan SSL-5L and a humbucker Seymour Duncan SH-4 JB (Seymour Duncan, Santa Barbara, CA, USA). Both pickup configurations were employed to observe the differences in their measurement results. A humbucker consists of two coils wired in opposite polarity and positioned next to one another. This configuration cancels electromagnetic interference; however, its wider sensing aperture acts as a low-pass filter, averaging the string’s motion over a larger area, which typically corresponds to a warmer perceived tone. In contrast, a brighter, more articulate sound is typically associated with single-coil pickups. It is not feasible to mount both pickups in the same position and orientation; therefore, they were left in their separate mounting locations, and measurements were conducted over both pickups.

**Figure 2:**
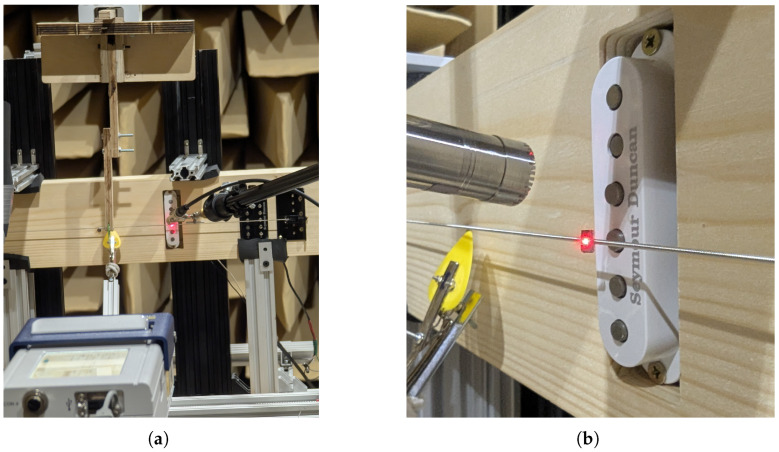
Alignment of (**a**) excitation mechanisms and (**b**) measurement sensors.

All measurement instruments were recorded using a National Instruments NI 9234 input module (National Instruments Corporation, Austin, TX, USA) with a sampling rate of 51,200 Hz. The positioning of all measurement methods is shown in Figure 2.

### 2.3. Excitation Mechanisms

**Figure 3:**
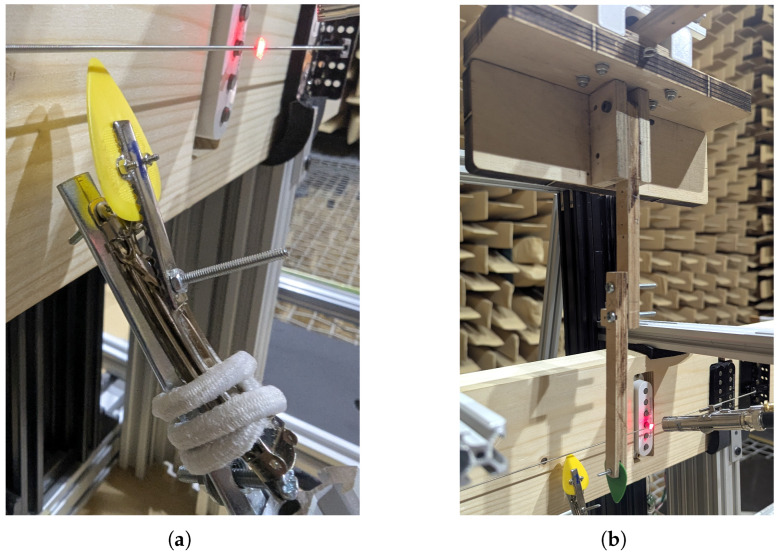
Constructed excitation mechanisms: (**a**) Plucker using a guitar plectrum mounted to a spring to mimic a guitar player. (**b**) Striker using a sideways-mounted guitar plectrum, mounted to a pendulum.

In order to eliminate string excitation as a variable, specialized mechanisms and solutions were designed and prepared for each excitation method. The first mechanism was designed to replicate a guitarist’s use of a plectrum. This requires the plectrum to strike the string with an initial velocity and continue its movement along an arc trajectory until the string slips off the plectrum and rings out. The mechanism performs this through incorporating a leaf spring from a hair clip, connected to a hinge on one side and a latch on the other. When closed, the spring is under tension and bounces away upon release. By attaching a nylon guitar pick to the free end of the spring, this motion can be used for the repeatable plucking of a string. A hair tie is used to catch the spring and dampen its vibration. This method of string excitation has been demonstrated to be repeatable in previous research [4,19]. The constructed mechanism is illustrated in Figure 3a. More advanced automated plucking robots have been utilized to achieve higher levels of plucking repeatability [20,21]; however, such a solution was impractical due to spatial limitations, the required direction of plucking away from the guitar body, and the need for an unobstructed path from the vibrometer to the string.

The second method of string excitation aims to provide a more defined, isolated pluck. A pluck requires the displacement of the string followed by its release. To achieve this, a copper wire is used. The wire is looped through the string at the excitation point and slowly pulled away from the instrument body. At a certain point, the wire breaks, releasing the string and allowing it to vibrate. Due to the repeatability of the copper wire, this method has previously been used for guitar string plucking [22,23] and has demonstrated a high level of repeatability [24]. In our experiment, the wire was pulled manually. Although automation of this process is possible for enhanced repeatability, the most common solution employs a solenoid [24]. However, this method is not viable when using electromagnetic guitar pickups, as the pulling coil generates significant electromagnetic interference, disrupting measurements.

The final method induces string vibration through a striking action. This is implemented using a pendulum mechanism with a nylon plectrum mounted perpendicular to the string. The pendulum is displaced to a predetermined angle and then released, allowing it to accelerate under gravity and strike the string with the edge of the plectrum. After impact, the pendulum rebounds and can be caught to prevent unwanted noise or interference with the string’s vibration. The edge of the plectrum serves as the contact point to minimize the contact area with the string, thereby reducing damping effects on the induced vibrational modes. The constructed pendulum mechanism is illustrated in Figure 3b.

All excitation mechanisms were configured to induce initial vibrations at the same point and in the same direction, perpendicular to the guitar body, aligning with the measurement direction. Since the focus of this research is on sensor technologies rather than the differences between excitation methods, the methods were not normalized to one another. To ensure the comparability of recorded results between methods, a string excitation normalization methodology would be required [25]. The absence of such a method renders direct comparisons of amplitude and spectral content between excitation types impossible.

### 2.4. Experimental Procedure

**Figure 4:**
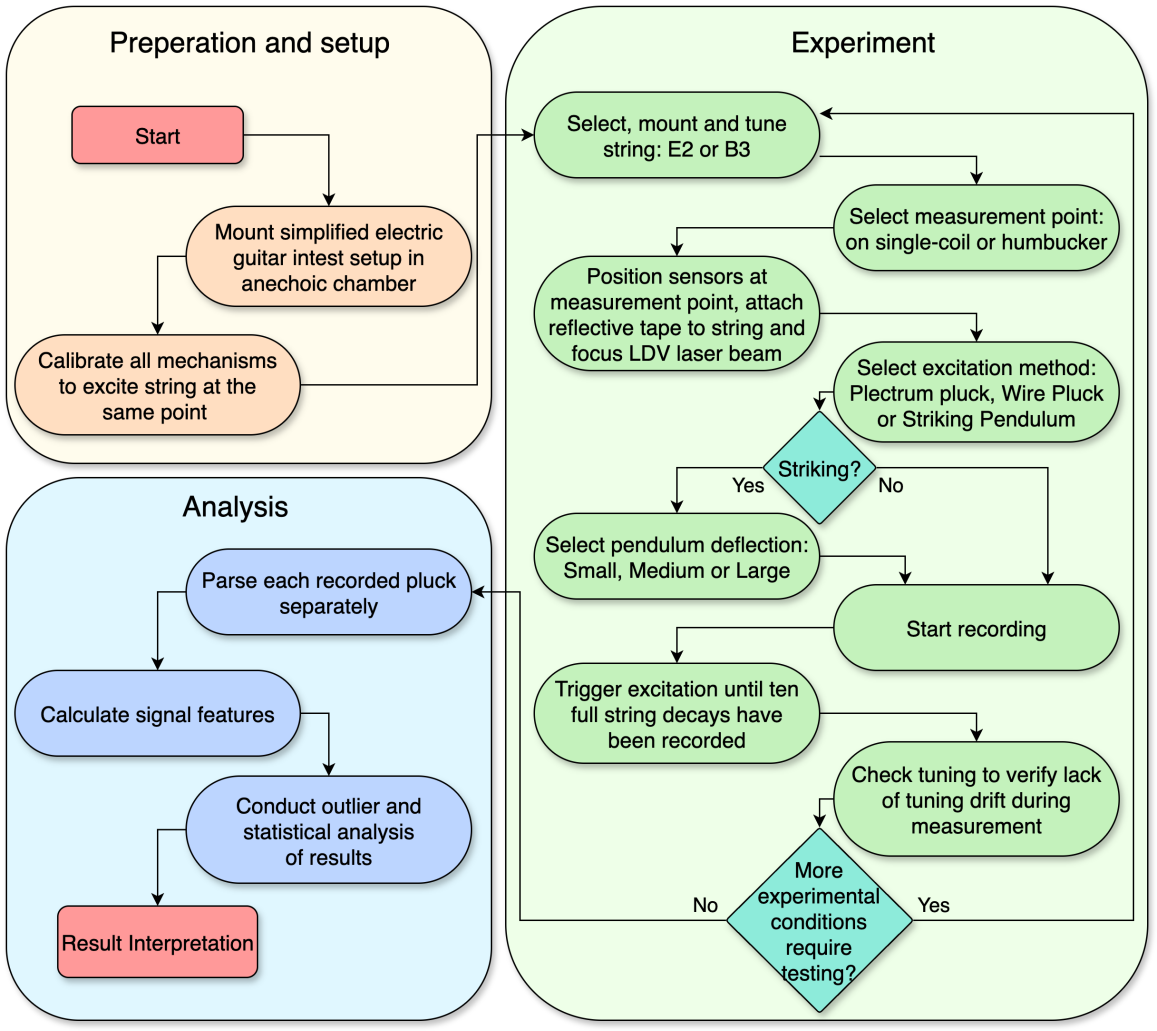
Diagram showing the experimental procedure including preparation, experimental recording, and data analysis.

The various components of the experimental method described in the preceding sections are brought together in the procedural flowchart shown in Figure 4. The diagram outlines the research process, from the initial preparation of the experimental setup to the iterative data acquisition loop, and the final stages of signal processing and analysis.

## 3. Comparison of Sensor Performance

The focus of this paper is the capacity of each sensor to accurately record string vibrations, with particular emphasis on the initial phase of excitation. Thus, we will begin the comparison of each technology by investigating their ability to record specific phenomena. This will show their practical usability in the task of precise waveform analysis of a guitar string.

### 3.1. Sensor Differences Overview

**Figure 5:**
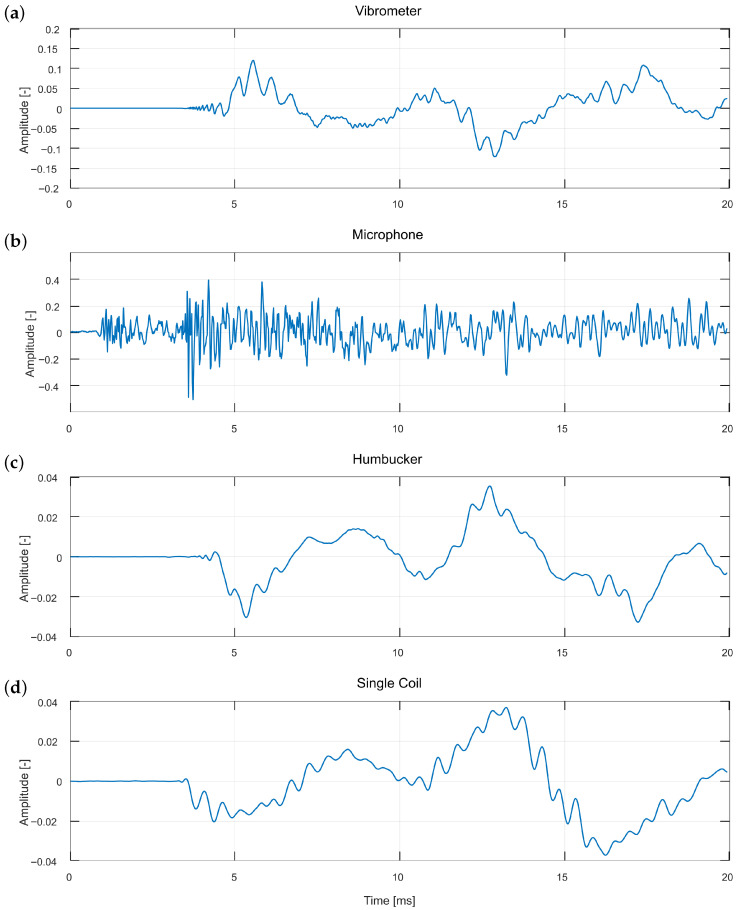
Close-up waveform comparison of an initial phase of the string plucking excitation, obtained using the plucker with a plectrum measured over the humbucker, for all four recording methods: (**a**) vibrometer, (**b**) microphone, (**c**) humbucker, and (**d**) single coil. The single coil measurements are not conducted in the same place along the string.

**Figure 6:**
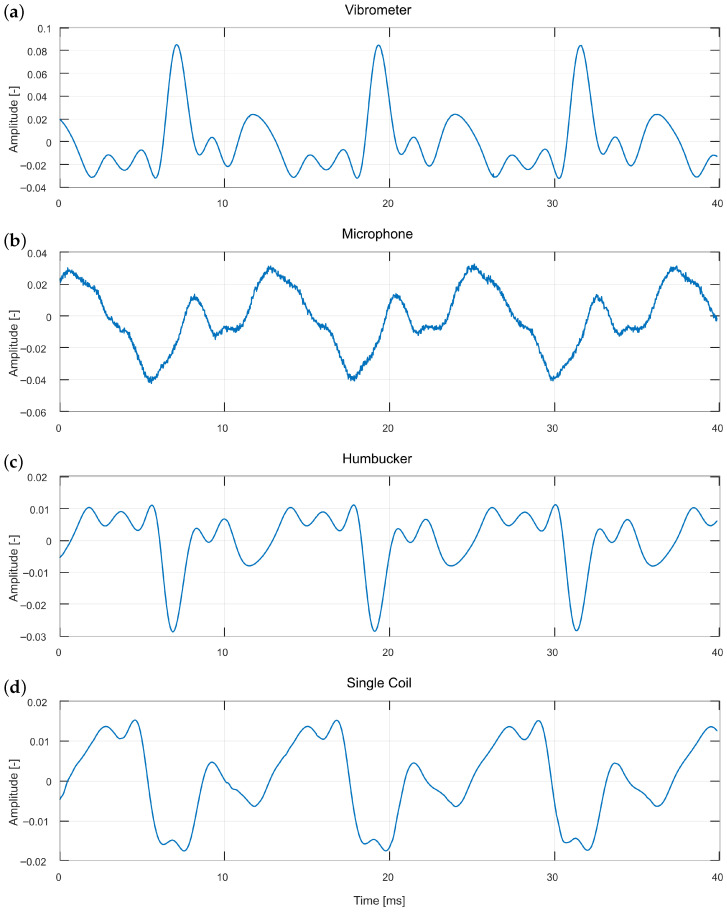
Close-up waveform comparison of the string plucking excitation, obtained using the plucker with a plectrum, two seconds after the pluck measured over the humbucker, for all four recording methods: (**a**) vibrometer, (**b**) microphone, (**c**) humbucker, and (**d**) single coil. The time scale is doubled compared to Figure 5. The single coil measurements are not conducted in the same place along the string.

The plucker mechanism equipped with a guitar plectrum (Figure 3a) provides a string excitation method that most closely resembles the actual playing conditions of a guitar. For this reason, it was selected as the starting point of the analysis. Figure 5 illustrates the moment of plucking as captured by all four sensors, whereas Figure 6 presents a segment of the same sound event two seconds after the initial pluck.

The first observation concerns the signal characteristics captured by each sensor. The microphone recording differs markedly from the other waveforms and also exhibits an earlier onset which is clearly visible in Figure 5, where the microphone signal precedes the responses of the other sensors by several milliseconds. This occurs because the microphone is the only sensor that detects air vibrations rather than the string itself. In the initial phase of the sound event (Figure 5), it registers the impulse-like noise generated by the excitation mechanism, which obscures the actual string vibrations. At this stage, no fundamental period is visible, and the waveform exhibits a noise-like character. In later stages, however, the signal becomes periodic, as illustrated in Figure 6. Combining microphone data with that obtained from any of the three remaining sensors may therefore provide a means of separating the excitation mechanism’s sound from the string vibration. The gradual transition from a noise-like to a periodic signal is visible in the comparison between Figure 5 and Figure 6, reflecting the decay of the excitation mechanism’s transient.

The vibrometer signal is inverted relative to the two guitar pickups, since they measure string vibrations from opposite sides. Although their waveforms appear inverted, they remain consistent in timing, demonstrating that the sensors are phase-aligned apart from this polarity difference. A notable difference concerns the level of detail present in the waveforms. The microphone retains most of the high-frequency components, though many of these likely originate from the excitation mechanism rather than the string. During the initial phase of the sound event, the vibrometer and both pickups produce very similar waveforms, with the vibrometer exhibiting the greatest level of detail, followed by the single-coil pickup, and the least from the humbucker. This order is consistent with the humbucker’s inherent low-pass filtering properties. After two seconds, this similarity significantly diminishes, with only the humbucker and vibrometer exhibiting comparable waveforms. The single-coil diverges from the vibrometer and humbucker signals, displaying mixed traits that partially resemble the microphone, suggesting a progressive loss of correlation between sensors over time. Although the microphone signal has become periodic, it superficially resembles the vibrometer signal, potentially due to measuring a different physical phenomenon or from the slightly different measurement perspective, as described in the experimental setup. The single-coil pickup waveform displays mixed characteristics of both the vibrometer and the microphone, which may in part also be attributed to the fact that the single coil measurements were not obtained at the same position along the string, as noted in the figure captions.

### 3.2. String Excitation When Striking

When a string is struck with a plectrum, the plectrum remains in contact with the string until the point of maximum displacement, after which the string rebounds and pushes the plectrum away. Consequently, the resulting vibration can be divided into two phases: (1) while the plectrum is in contact with the string and (2) after it has disengaged. In the first phase, the vibration is constrained not only at the string ends but also at the contact point. This effect has been theoretically described as the independent vibration of string segments, effectively creating shorter strings with correspondingly higher fundamental frequencies [26]. As a result, string vibrations during the onset are expected to exhibit a higher base frequency compared to the subsequent steady-state phase.

**Figure 7:**
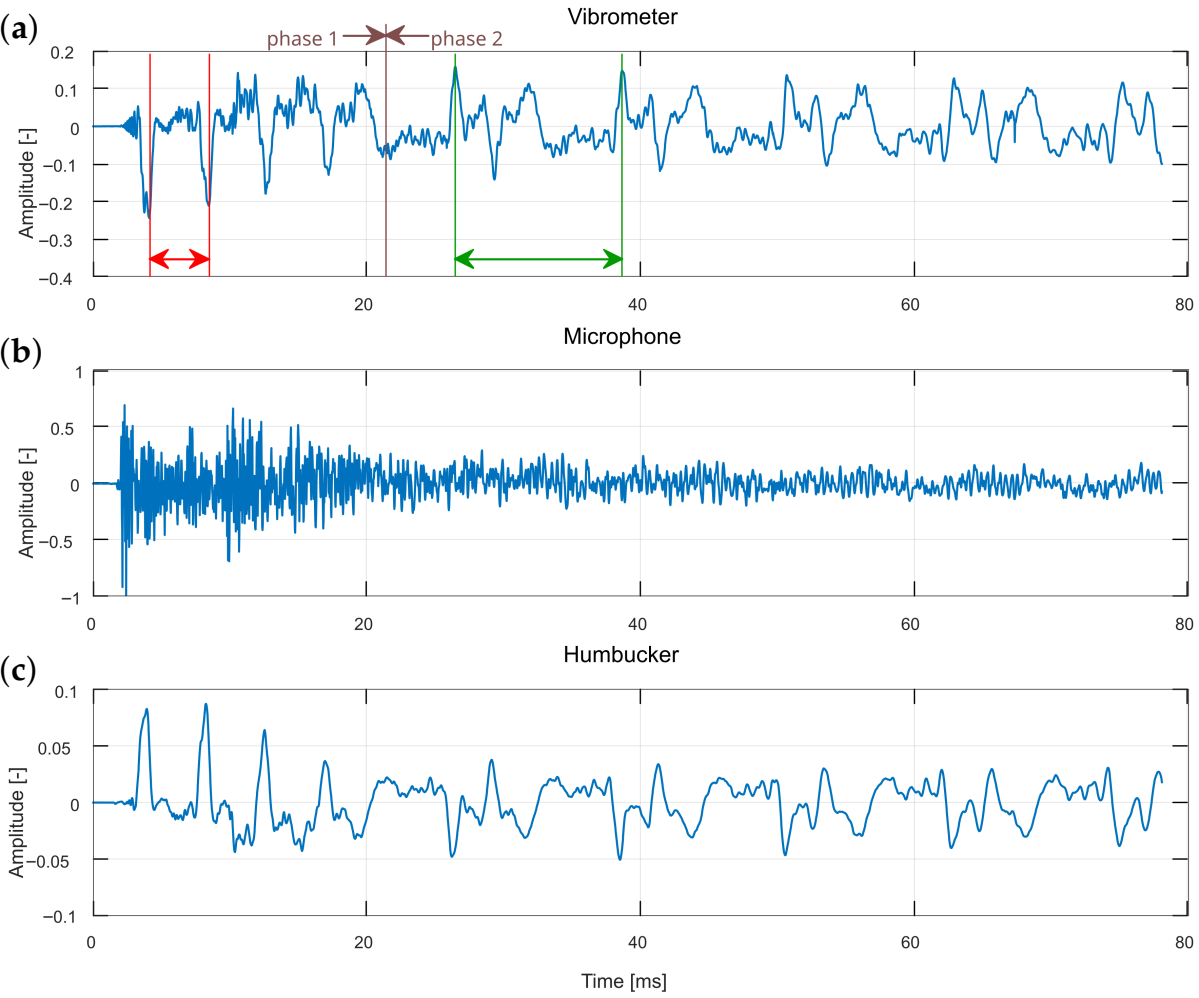
Comparison of striking excitation waveforms measured over the humbucker, for different recording methods: (**a**) vibrometer, (**b**) microphone, and (**c**) humbucker. Two phases can be observed as follows: 1—vibrations of a divided string segment, and 2—vibrations of the entire string. Red and green sections represent vibration periods in both phases.

**Figure 8:**
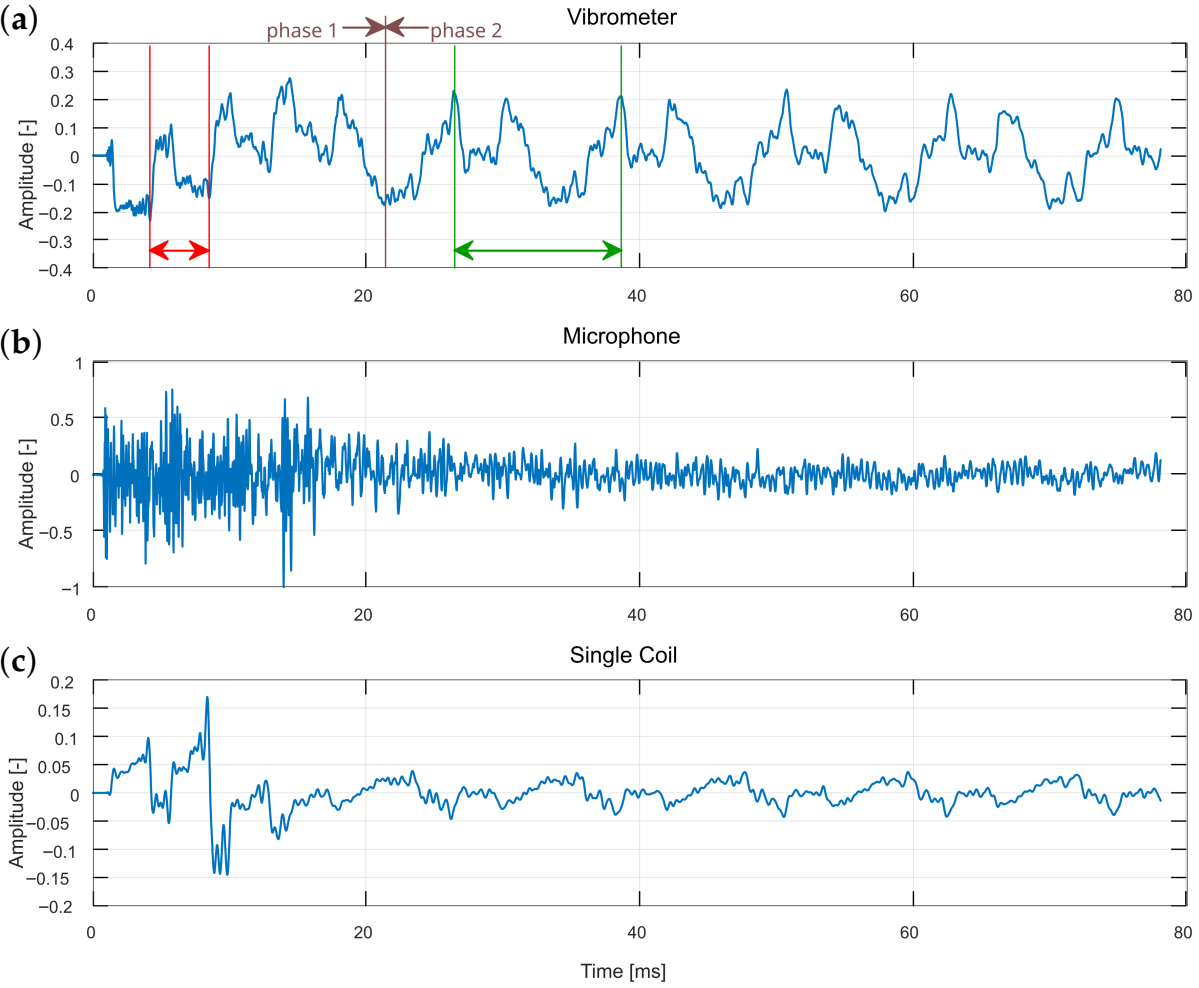
Comparison of striking excitation waveforms measured over the single coil, for different recording methods: (**a**) vibrometer, (**b**) microphone, and (**c**) single coil. Two phases can be observed as follows: 1—vibrations of a divided string segment, and 2—vibrations of an entire string. Red and green sections represent vibration periods in both phases.

Figure 7 and Figure 8 present waveforms recorded with different methods for two separate strikes, measured above the humbucker and the single coil, respectively. As noted earlier, the microphone signal is dominated by the excitation mechanism, which obscures the crucial initial phase of the event. By contrast, the vibrometer and humbucker clearly reveal the phenomenon of the string being divided by the plectrum: during the first 20 ms, four short vibration periods (indicated in red) can be observed before the signal transitions to the expected fundamental period of the entire string (indicated in green). In the single coil data, traces of the phenomenon are present, though the distinction between short and long periods is less pronounced than in the vibrometer and humbucker. It should be noted that although differences in measurement position (as in Figure 7 and Figure 8) could influence the visibility of the phenomenon, the vibrometer consistently detected it at both positions. This indicates that the poorer performance of the single-coil pickup is attributable to the sensor characteristics rather than the measurement location.

Another marker of this effect is visible in the vibrometer and humbucker signals: during the initial short periods, the waveform exhibits a gradual increase in amplitude, reflecting the continued displacement of the string by the moving plectrum until disengagement occurs. This amplitude modulation is not evident in the single coil or microphone recordings, further highlighting the advantage of certain measurement methods.

The ratio of the short-period oscillations to the final fundamental period provides an estimate of the excitation point on the string, which, in this case, corresponds to 0.359 of the total string length. The vibrometer data, characterized by a sharper and more detailed waveform, facilitates precise identification of periodic markers. In contrast, the smoother humbucker waveform may be more effectively analyzed using autocorrelation rather than relying solely on visual inspection of waveform peaks.

### 3.3. Plucking Position and Measuring Point Estimation

Several methods have been proposed for estimating the plucking and measurement positions of a string based on recorded signals. These approaches include autocorrelation of spectral peaks [27], parametric pitch estimation [28], and, when precise recordings of string vibrations are available, analysis of wave reflection timing [26,29]. By capturing the temporal evolution of the string’s transverse displacement with high precision, it is possible to examine the relative phase and amplitude content of its vibrational modes. The proportion and timing of reflected wave components provide sufficient information to infer the excitation position.

**Figure 9:**
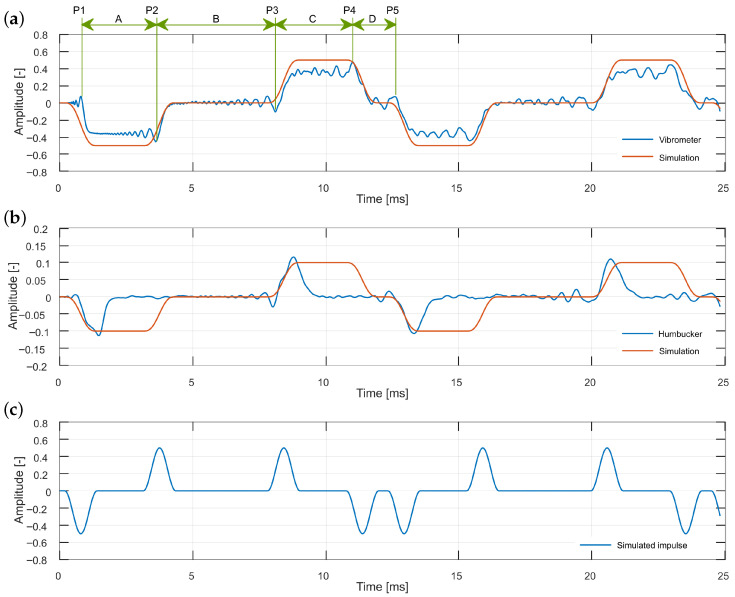
Analysis of plucking data obtained from vibrometer (**a**) and humbucker (**b**) observing string plucked by the copper wire, and from the finite-difference simulation (**c**). P1–P5 are subsequent impulses (straight or inverted, as shown on bottom, simulated plot) travelling over the measurement point. $T_{0}=A+B+C+D$ is a single vibration period representing the fundamental frequency. $\frac{B}{T_{0}}$ allows us to estimate excitation position, and $\frac{C}{T_{0}}$ estimates measurement point, both as a fraction of a string length.

Wave reflections are most clearly observable in cases of clean excitation; thus, plucking with a copper wire will be analyzed. To enable proper identification of waveform features as successive reflections, a finite-difference simulation of an idealized string was performed and compared to the experimental recordings, as shown in Figure 9. The simulation was based on the one-dimensional wave equation:

(1) $$\frac{\partial^{2}u}{\partial t^{2}}=c^{2}\frac{\partial^{2}u}{\partial x^{2}},$$

where u(x,t) represents the displacement of a string and *c* is the transverse wave velocity:

(2) $$c=\sqrt{\frac{T_{0}}{\rho A}},$$

where $T_{0}$ is the tension of a string, ρ is the density of the string, and *A* is the area of the cross-section of the string. The excitation and readout points in the simulation were adjusted to match those of the experiment.

The vibrometer recordings (Figure 9) correspond closely with the simulation results, allowing straightforward identification of all traveling impulses along the string. The fine temporal detail captured by the vibrometer enables accurate measurement of inter-impulse distances, yielding the results presented in Table 1. Among the tested sensors, only the vibrometer provides the level of precision required for such waveform analysis. The humbucker captures some, but not all, of the relevant impulses, while the single coil and microphone do not exhibit the necessary features.

## 4. Measurement Method Characteristic Comparison

In this chapter, we will focus on a more general comparison of the sensors and their characteristics.

### 4.1. Signal-to-Noise Ratio

A way to evaluate the performance of each measurement technique is to calculate signal-to-noise ratio. This can be achieved through isolating segments of the recording in which there is no vibration of the string and comparing it to segments after plucking the string. The beginnings of plucks were located. Two second segments before and after these moments were cut to constitute the noise and signal signals. Plucks containing transient noise prior to string excitation were excluded from the analysis. To ensure comparability, all methods used the same signal segments for calculation. The signal-to-noise ratio (SNR) values were calculated using the formula:

(3) $${\mathrm{SNR}}_{\mathrm{dB}}=10\log_{10}\left(\frac{P_{\mathrm{signal}}+P_{\mathrm{noise}}}{P_{\mathrm{noise}}}\right)$$

**Figure 10:**
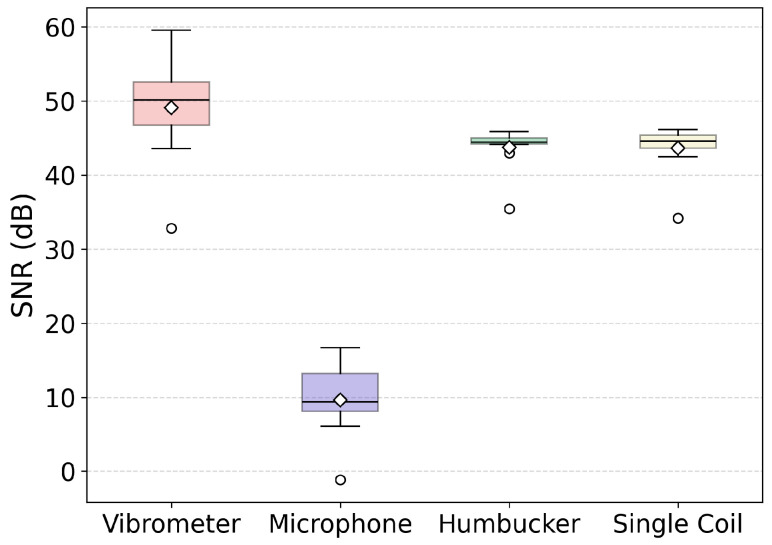
Comparison of SNR values calculated for different recording methods across N = 10 recorded strikes. Mean, median, IQR, typical values, and outliers are presented. Note: The humbucker measurements are not conducted in the same place along the string.

In Equation (3), $P_{\mathrm{signal}}$ is the power of the signal segment and $P_{\mathrm{noise}}$ is the power of the noise segment. Given that the same level of background noise was present during the recording of the signal, the form of SNR that sums both powers in the numerator was selected as more appropriate. Figure 10 shows a comparison of SNR values calculated for each recording method.

A few conclusions can be drawn from these results. The most important is the much lower values calculated for the microphone. This is due to two factors. Firstly, this reflects the inherently weak acoustic radiation of a single unamplified string in free air. Since the vibrating string is not amplified by any mechanical system, such as an acoustic guitar body, the signal level is low. Secondly, despite recording in an anechoic chamber, the microphone is still susceptible to noise generated by the operation of the plucking mechanisms. This shows the clear advantage of the optical and electro-acoustic methods in this experimental use case. The anechoic chamber provided both vibrational isolation and a low level of electric interference leading to high SNR values for these methods. The similar levels achieved by the single coil with 43.66 dB (SD = 2.9, N = 10) and humbucker with 43.74 dB (SD = 2.9, N = 10) suggest that the background electromagnetic interference was very low, rendering the interference cancellation provided by the humbucker unnecessary. Nonetheless, this factor should be considered when recording in less optimal environments. The highest SNR values were recorded for the vibrometer at 49.1 dB (SD = 6.9, N = 10); however, the advantage over the magnetic pickups was not substantial.

### 4.2. Response to Varying String Excitation Levels

An aspect worthy of investigation is how each method captures the differences induced by variations in the initial striking speed. To this end, signals were recorded for three different initial pendulum deflection. To ensure the statistical reliability of the findings and to assess the reproducibility of each measurement, a total of N = 10 trials were recorded for each experimental condition. The strikes were recorded using a 240 frames-per-second video camera, and the velocity of the plectrum at the moment of striking was calculated through frame-by-frame analysis using the angular displacement and the length of the arm. The results are presented in Table 2.

**Figure 11:**
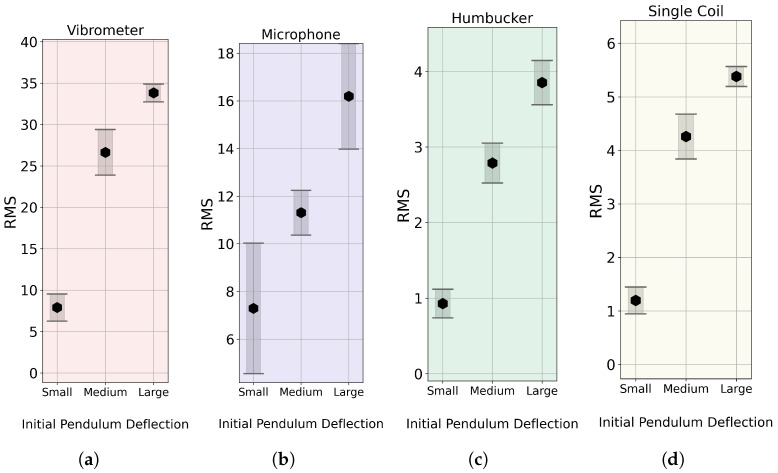
Comparison of average RMS of recorded signals for different initial pendulum displacements by different methods: (**a**) vibrometer (**b**) microphone (**c**) humbucker pickup, and (**d**) single coil pickup. All results are presented based on N = 10 trials, with the mean and standard deviation presented. Note: The humbucker measurements are not conducted in the same place along the string.

To compare the sensors, each pluck was extracted with 0.5 s of time before the strike and 7 s of decay. The Root Mean Squared (RMS) values were calculated and averaged within each measurement series. The results obtained are presented in Figure 11. The focus is not on the absolute values but on the overall shape, which illustrates how each sensor records signals at varying dynamic levels. Consequently, the y-axis limits have been set so that the top and bottom positions for each sensor align at the same point.

The obtained results demonstrate clear differences in the dynamic response and linearity of the sensors. It is important to note that a linear increase in striking velocity does not necessarily correspond to a linear increase in the generated string vibration energy. These results do not allow for the assertion of which shape is the most accurate and may only be used for comparative purposes. All methods successfully registered increased signal energy with higher striking velocity, as indicated by the non-overlapping standard deviation bars for each of the three dynamic levels across all sensors. The increases recorded by the vibrometer and the single coil exhibit very similar shapes. In contrast, the humbucker demonstrates a more linear shape, while the microphone is a clear outlier, as its results curve in the opposite direction. This indicates that the measurement methods possess different dynamic characteristics, with the vibrometer and single coil being the most similar. Lastly, since all methods effectively resolved the three dynamic levels, we can conclude that they all possess a sufficient dynamic range for typical guitar analysis.

### 4.3. Filter Transfer Functions

**Figure 12:**
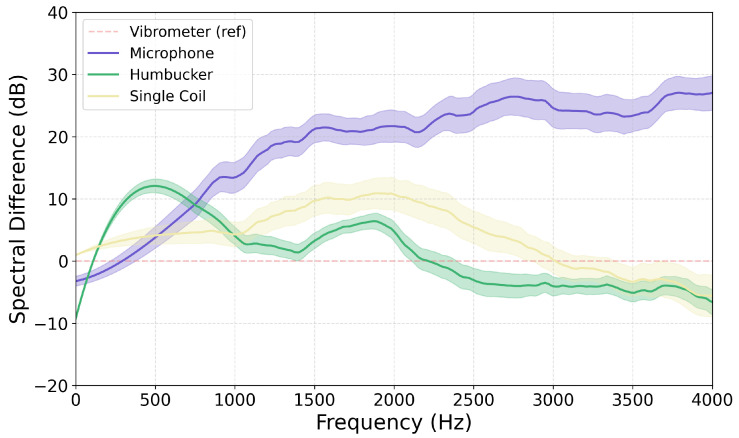
Comparison of smoothed average Filter Transfer Functions calculated for each method using the vibrometer as the reference. All values calculated across N = 10 strikes with the solid line representing the mean value and the shaded area showing standard deviation. Note: The humbucker measurements are not conducted in the same place along the string.

The next step was to visualize the spectral difference between the signal recorded between each method. To this end, the Filter Transfer Function was calculated for each method in relation to the vibrometer. It was chosen as the baseline due to the high quality of results it produced together with low noise. Recordings conducted for the same plucks were cut to the first two seconds, normalized to −23 LUFS [30], and their transfer functions were calculated and smoothed. This was performed for three strikes conducted using the medium setting of the pendulum. The achieved results are presented in Figure 12.

The functions obtained for both electromagnetic pickups are much closer to the results acquired with the vibrometer than those obtained from the microphone. This difference for the microphone increases with rising frequencies, likely due to a combination of captured noise captured and the influence of frequency-dependent acoustic radiation from the string. The characteristics of the pickups exhibit notable similarities, with the primary distinction being the poor performance of the humbucker at frequencies below 1 kHz and the single coil in the 1–3 kHz range, where values exceed 10 dB. Such a comparison between the magnetic pickups is not unexpected, as it aligns with previous theoretical and experimental research [31,32] which often frames a humbucker as having a low pass characteristic. The two coils wired in series in the humbucker increase the total inductance, capacitance, and resistance of the circuit. This, in turn, lowers the resonant frequency and reduces the Q-factor of the pickup’s RLC circuit. The higher inductance impedes high-frequency current changes, while the increased resistance dampens the resonance. This results in an attenuation of high frequency content. Additionally, the wider magnetic aperture of the humbucker is sometimes mentioned as averaging string motion over a larger region, further filtering out higher spatial harmonics. It is worth noting that both magnetic pickup designs perform poorly at higher frequencies, with their frequency response functions deviating further from the reference vibrometer as frequencies increase.

## 5. Influence of the Reflective Tape on the String’s Vibration

**Figure 13:**
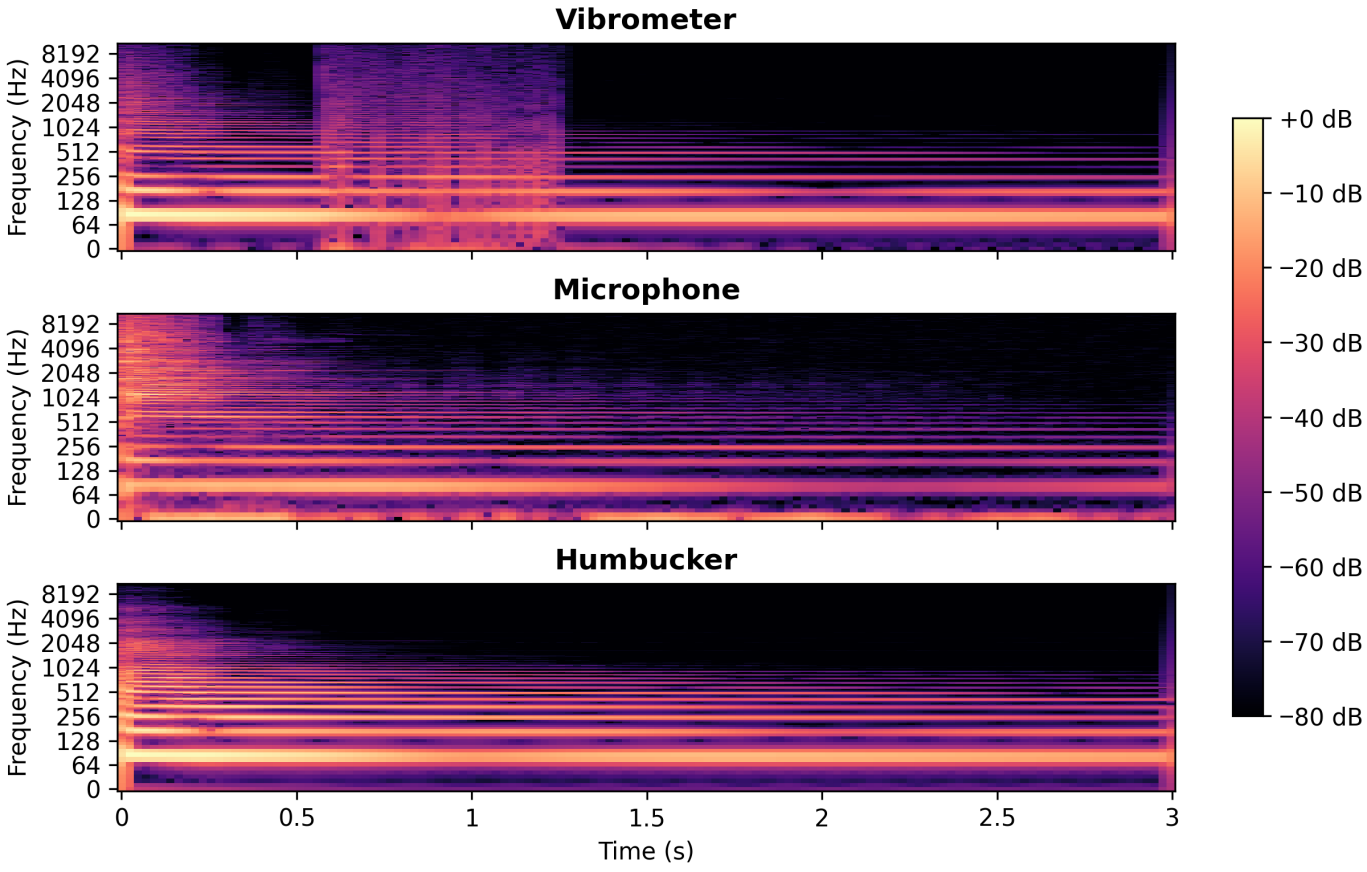
Spectrogram of pluck conducted with 0.014 mm diameter wire recorded with vibrometer, microphone, and humbucker.

As described in Section 2.2, measurement of string vibration using the vibrometer required the use of a reflective tape affixed to the string. Without this tape, the string would move out of the laser’s path, resulting in artifacts and inaccurate measurement results. This issue occurred despite attempts made while measuring the thicker string, with the excitation direction parallel to the laser’s direction. The use of this tape does; however, raise the question of its influence on the measured string vibration. The smallest tape size that yielded accurate results was 3 × 4 mm. Despite using this tape, when plucking the string with a 0.14 mm diameter copper wire, the string’s sticker moved out of the laser’s path. This constitutes a strong pluck, exceeding the dynamics of regular guitar playing techniques. It illustrates issues that would arise at lower levels if a smaller reflective tape size were used. A spectrogram of the recording of this pluck using different methods is presented in Figure 13.

When analysing the spectrogram of the vibrometer recording between 0.5 and 1.3 s, broad spectrum interference is present in the signal. This issue is not present in the recordings conducted using the microphone and humbucker, indicating that it is not a recording of a true phenomenon in the string’s vibration. This noise is caused by the string vibrating at a magnitude which results in the sticker leaving the lasers path and thus making the resulting recording unusable. It is also worth noting that this happens despite the string being initially plucked in the direction of the laser. This is the reason why this noise is not present from the beginning of the pluck despite the string’s vibration magnitude being highest at that moment. The string begins its vibration in the direction it was plucked, but as the vibration decay progresses, this plane of vibration dissolves into vibrations in both transversal polarizations [6]. These results show that the size of reflective tape cannot be further decreased to reduce weight.

Weighing the whole sheet, calculating the area density, and multiplying it by the area of the used 3 × 4 mm tape yields a weight of 0.0056 g. Comparing this weight to the effective segments of the thicker and thinner strings, 4.4 g and 0.3 g, respectively, demonstrates how this additional weight can pose issues when measuring thin strings. Lighter reflective tapes were also tested but their lack of rigidity caused them to bend and sway when affixed to the string resulting in incorrect measurements. It should also be noted that the sticker can influence the string’s vibration not only through weight but through the increased resistance when moving against the air during vibration.

**Figure 14:**
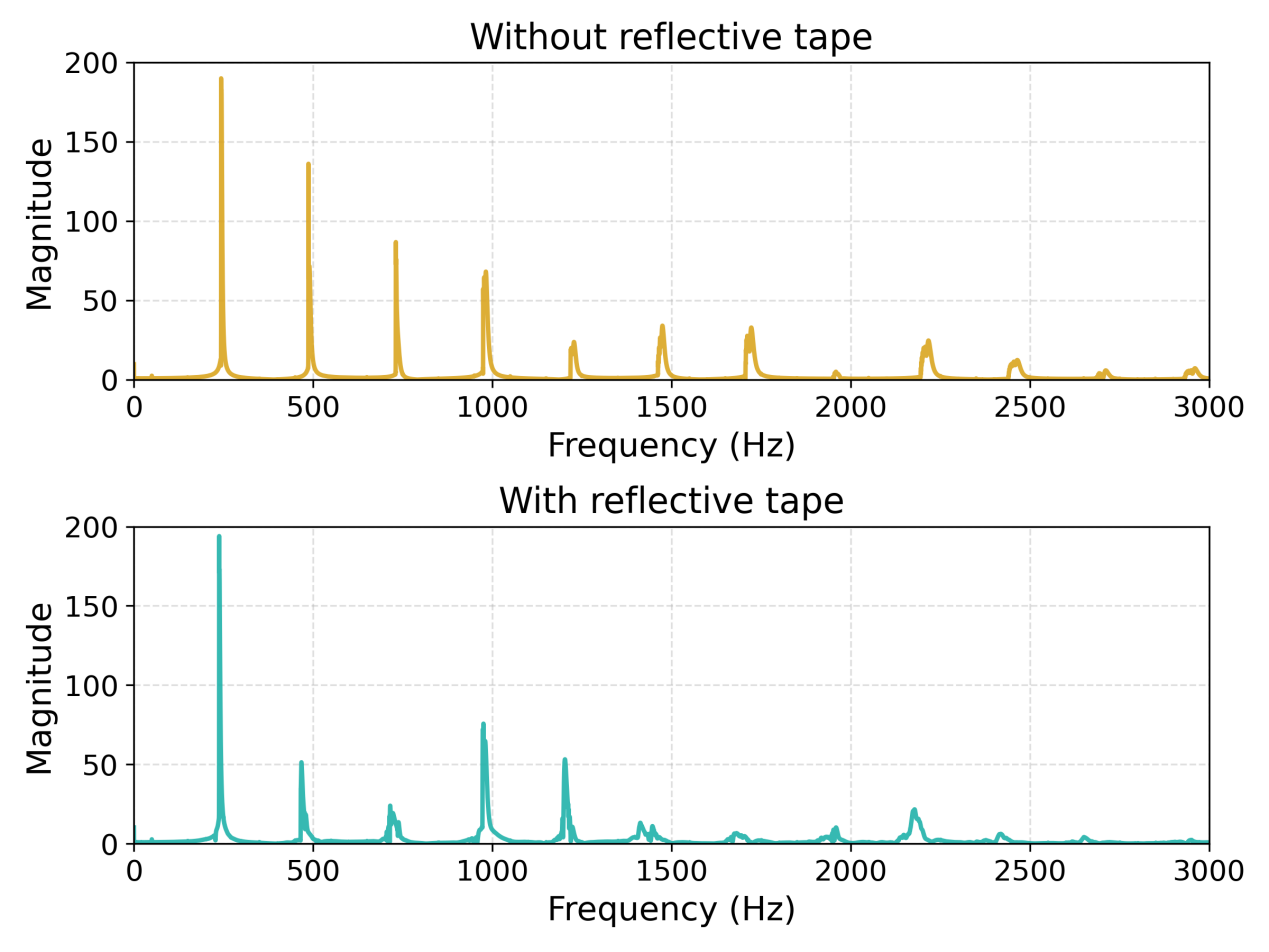
Magnitude spectrum comparison of wire plucks recorded using the humbucker for the 0.28 mm string without and with the vibrometer reflective tape adhered to the string.

To show this measurement, recordings were conducted using the humbucker, with the thinner 0.28 mm string being plucked using a wire. Measurements were conducted without and with the reflected tape added to the string. A comparison of the achieved spectrums is shown on Figure 14.

**Figure 15:**
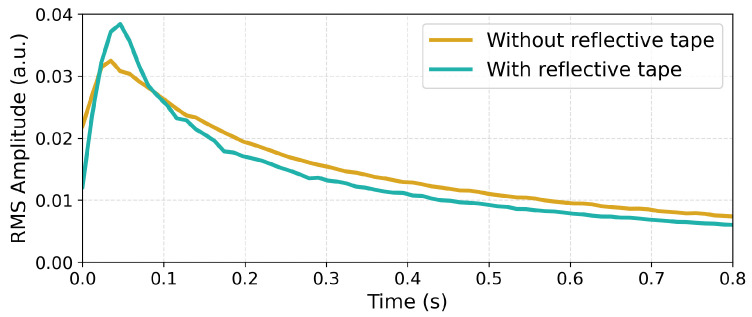
RMS envelope comparison of wire plucks recorded using the humbucker for the 0.28 mm string without and with the vibrometer reflective tape adhered to the string. “a.u.” denotes “arbitrary units”.

The influence of the sticker is clearly evident, resulting in significant damping of certain harmonics and amplification of others. This is particularly visible in the 400–800 Hz and the 1400–1800 Hz ranges. The impact is also visible in the time envelope of the recorded sound. A comparison of RMS envelopes is shown on Figure 15.

The additional mass clearly influences the temporal characteristics of the string’s vibration. While the initial pluck is higher, the reflective tape introduces additional damping, evident in the faster decay of the blue curve.

These differences exceed the limits of imperceptibility, and such an influence on the measured phenomena is unacceptable for a measurement method. This indicates that, while laser vibrometry can achieve high precision, its potentially invasive nature, when requiring a target, presents a significant and sometimes prohibitive trade-off for lightweight structures such as guitar strings. It is important to note that a string gauge of 0.28 mm (0.011 inch) is realistic, as typical electric guitar sets have a high E string ranging from approximately 0.20 to 0.3 mm (0.008–0.012 inch).

### Measuring the Influence the LDV Optical Target Tape Has on a String’s Vibration

**Figure 16:**
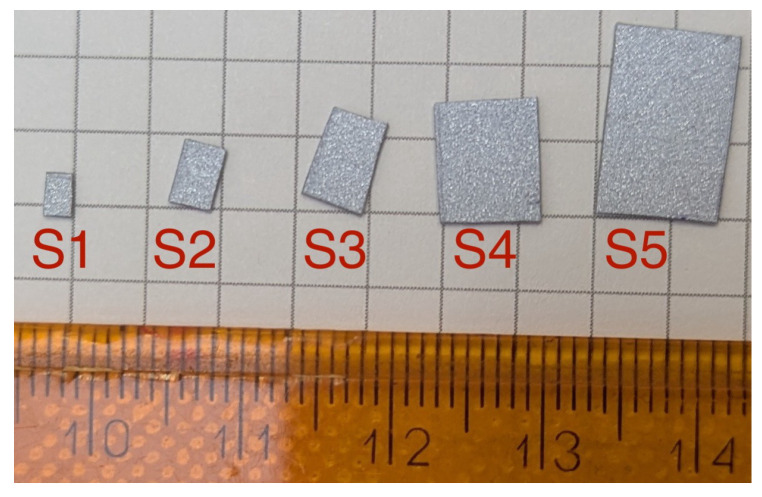
Reflective stickers used as optical targets for comparison. The labels S1–S5 indicate the configuration names assigned to each sticker.

To better understand how the use of a vibrometer reflective sticker affects string dynamics, a series of experimental measurements were conducted. The experimental setup was identical to the previous recordings, with the exception of the string, which was a 1.12 mm (0.044 inch) with an active weight of 4.14 g, tuned to E2 (82.41 Hz). The vibration of the string was measured using all recording methods for an empty string (NoS) and with reflective stickers of various sizes attached to the string at the measurement point. Figure 16 shows the used fragments of reflective tape, and Table 3 presents a comparison of their parameters.

**Figure 17:**
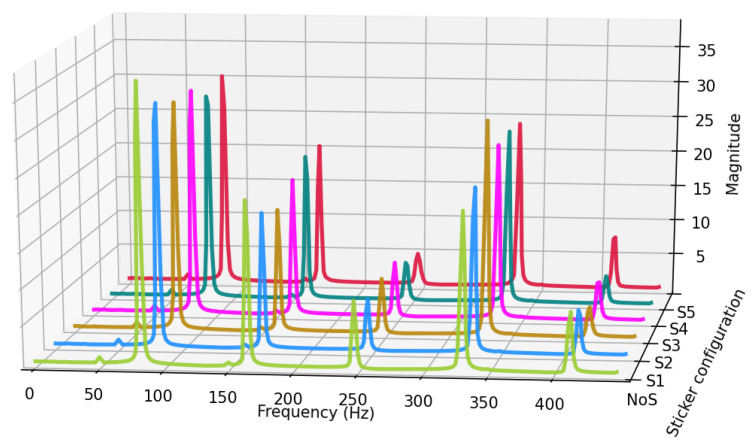
Average spectra of excitation series obtained from manual wire plucking, recorded with the humbucker for each sticker configuration. Results are averaged over N = 10 trials.

The first aspect worth investigating are the spectrums of the recorded signals. Figure 17 presents a comparison of spectra from a single excitation recorded for each tape configuration.

**Figure 18:**
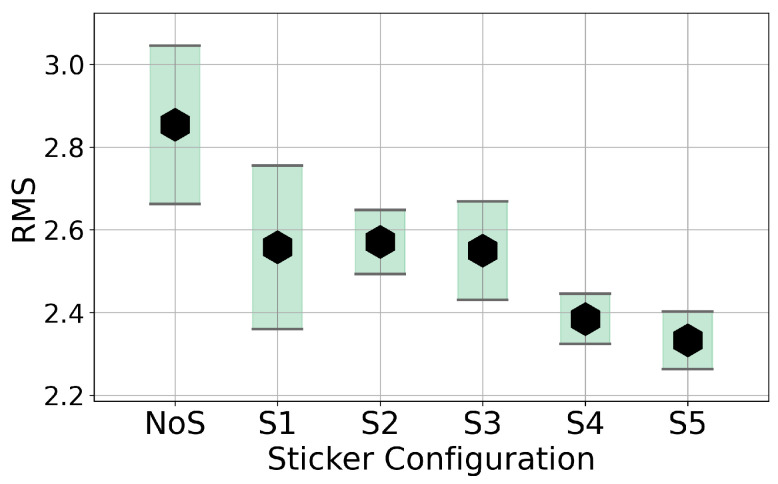
Average RMS values and standard deviations of humbucker recordings for wire plucking with various sticker configurations on the string. Results are averaged over N = 10 trials with the mean and standard deviation shown.

Analysis of the recorded, averaged spectra indicates that even the addition of the smallest sticker, S1, significantly alters the proportions of the harmonics. A sizeable decrease in the magnitudes of the first and second harmonics is observed, while the third and fourth harmonics are diminished to a lesser extent. As the tape size increases, this tendency remains but a clear characteristic of change does not manifest. This suggests that the sticker’s effect is not purely mass-related but may depend on complex modal and aerodynamic interactions. Certain partials can be significantly affected, such as the fourth harmonic in S2 and the fifth harmonic in S5, which are notably stronger than for other stickers. A measurable shift in the string’s base frequency was not observed for any sticker size. In the absence of a discernible trend, it is worthwhile to investigate signal parameters. The first parameter is the RMS of the signal, as presented in Figure 18.

**Figure 19:**
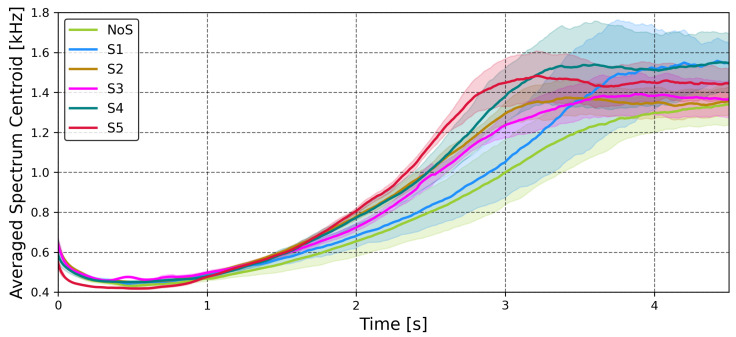
Average spectral centroid over time for wire plucking series, recorded with the humbucker for each sticker. Each configuration result represents N = 10 trials with the solid line representing the mean value and the shaded area showing standard deviation.

Analysis of the RMS clearly indicates that the addition of reflective tape consistently reduces the signal’s energy. This observation holds true even for the smallest size, S1. Notably, the change is not consistent, with S1, S2, and S3 exhibiting similar values, although measurement uncertainty makes this relationship unclear. Another approach worth analyzing is investigating to what range the addition of a sticker influences the timbre of the played note. To this end, Figure 19 shows the change of spectral centroid over time for different stickers. Spectral centroid is an important parameter to investigate as it has been shown to highly correlate with listener perception of a sound’s brightness [33].

These results show, that although all configurations exhibit similar values initially, they begin to diverge after the first second and demonstrate significant differences during the third second of sound decay. These differences increase from the NoS values as the size of the sticker increases.

These results clearly illustrate the influence of the reflective sticker on string vibration. The differences are measurable and exceed the limits of perceptibility [34]. They are evident in both temporal and spectral analyses, as well as in the timbral features of the signal. This influence is observed even at a sticker size that is too small to provide a reliable optical target for the LDV, resulting in artifacts in the recorded signal. These results lead to the conclusion that when measuring instrument strings, LDVs cannot be used as a non-contact measurement method that does not influence the measured object.

This magnitude of change occurs despite the tape weighing only 0.1% of the string’s mass. Several factors may contribute to this phenomenon. Firstly, the mass added by the sticker is concentrated in a small area. When converting the masses of the string and tape to linear density, the tape can constitute up to 10% of the string’s density at that point. This means that it can disrupt the linear distribution of mass along the string, altering vibration modes and diminishing harmonicity. Secondly, the sticker adds not only mass but also surface area in the direction of initial excitation. This results in increased drag on the string, which enhances damping. Once again, this increase is localized, potentially disrupting the string’s natural vibration. Thirdly, due to imperfect mounting, the sticker is not perfectly symmetrical on the string. Additionally, it is not perfectly stiff. These factors may cause evolving changes in vibration polarisation and type.

## 6. Discussion

The experimental results indicate that the choice of measurement technology is not neutral and involves significant trade-offs between precision and practicality. Each sensor captures a distinct aspect of the string’s vibration, and no single method can be regarded as universally superior. Therefore, the selection is dependent on the specific research setup and question.

The laser vibrometer is considered the standard for non-contact, high-precision measurement. Its primary advantage is its ability to directly measure the mechanical motion of the string, providing exceptional immunity to confounding factors such as instrument body resonance and ambient acoustic noise. Inherent limitation to a single axis of measurement can be beneficial for analyzing the polarization modes of the string’s vibration. Moreover, its designation as a measurement tool ensures a high level of reliability and repeatability. Nonetheless, this advantage is accompanied by a significant monetary cost. The method is sensitive to setup, requiring precise targeting of the laser on a reflective point. Critically, our results indicate that measuring a string’s vibration requires a reflective sticker. This tape influences the string’s vibration and renders the measurement of thin strings impossible. Even for thicker strings, the influence of the additional weight added to the string must be accounted for. This limitation needs to be recognized during experimental planning. The need for precise targeting also makes it highly impractical for studies involving human musicians, as any movement of the instrument renders the measurement unstable, confining its use to highly controlled laboratory settings.

In contrast to the vibrometer’s isolated focus, the microphone offers a more general perspective on the vibration. It is also a specialized and calibrated measurement tool. It does not isolate the vibration of the string, being susceptible to the vibration of other elements, sound created by the test procedure and outside noise. The measurement of air vibration generated by the string instead of the string itself adds a level of delay and filtration. This means that the microphone is not adequate when conducting precise waveform analysis. It captures the holistic acoustic event as a human ear would perceive it, making it the most valid method for perceptual or psychoacoustic research.

Magnetic pickups offer a pragmatic compromise. They are low-cost, easy to position reliably, and share the vibrometer’s immunity to ambient acoustic noise. However, they are susceptible to electromagnetic interference. They require ferromagnetic strings to function and, more importantly, act as inherent filters that influence the signal. No guitar pickups are designed for measurement purposes with a flat frequency response. Despite this, our measurement results show their ability to register precise waveform events at a level similar to the vibrometer.

### Sensing-Method Selection Based on Application Requirements

When designing a measurement setup, the stated experimental goal will dictate which of the tested technologies can be used. The presented results can be employed to establish a practical set of systematic guidelines concerning use cases, strengths, and limitations that must be considered during experiment planning. Table 4 presents a comparison of measurement methods and what investigations they are appropriate for based on our results and analyses.

Additionally, sensor technology limitations mean that not all methods are possible to use depending on the requirements specific to every experimental situation. Table 5 presents which methods can be used depending on measurement scenarios.

To assist experimental design, the main takeaways regarding each measurement technology have been summarized in Table 6 for easy referencing.

## 7. Conclusions

In this study, three sensor technologies—microphone, magnetic pickups, and laser Doppler vibrometry (LDV)—were compared for their ability to capture the vibration of a guitar string under controlled excitation. The results showed that the choice of sensor strongly affects which aspects of string motion can be observed during the transient and decay of string motion.

The experiments confirmed that no single sensor can be considered universally superior. Instead, each method was found to be best suited to different research contexts. Laser Doppler vibrometry (LDV) offers unmatched precision in controlled laboratory studies; however, it necessitates an optical target, which, despite its low weight, influences the string’s vibration. This influence is measurable in temporal, spectral, and timbral analyses. It must be taken into account when analysing results and renders the LDV ineffective for thin strings. Magnetic pickups provide a pragmatic balance of accuracy and usability for applied guitar research at a low cost. They allow precise waveform analysis but their inherent signal filtering must be taken into account when conducting more general timbre analysis. Microphones remain most relevant when perceptual or psychoacoustic questions regarding a full instrument are of interest rather then precise measurement of a single element vibration. In this configuration, the microphone did not provide sufficient precision for string vibration analysis.

Future work should address limitations of this study by exploring lighter or non-invasive optical markers, quantifying their impact on the measured physical system, implementing fully automated plucking mechanisms which minimise both acoustic and electromagnetic interference, and investigating how non-ideal measurement environments and scenarios influence the usability of specific measuring solutions. Further studies of sensor fidelity in the time domain would also clarify their relative suitability for waveform-accurate modeling and synthesis.

Overall, this study demonstrates that the choice of sensing technology represents a crucial methodological decision in guitar acoustics, with significant implications for experimental design and applied research in musical acoustics, particularly in physical modeling.

## Figures and Tables

**Table 1:** Comparison of physical measurement and estimation based on signal locations of measurement and excitation points.

	Physical Measurement	Signal-Based Estimation
Excitation Location	$\frac{240\mathrm{mm}}{645\mathrm{mm}}\approx 0.372$	$\frac{(414-184)\mathrm{samples}}{604\mathrm{samples}}\approx 0.381$
Measurement Location	$\frac{157\mathrm{mm}}{645\mathrm{mm}}\approx 0.243$	$\frac{(564-414)\mathrm{samples}}{604\mathrm{samples}}\approx 0.248$

**Table 2:** Measured velocity of the pendulum at the moment of striking for various initial pendulum deflections.

Pendulum Deflection	Initial Deflection (°)	Measured Striking Velocity (m/s)
Small	37	0.49
Medium	75	1.12
Large	112	1.74

**Table 3:** Dimensions and weights of the reflective stickers used.

Configuration Name	Sticker Dimensions [mm]	Sticker Area [mm^2^]	Sticker Mass [g]
NoS	0 × 0	0	0
S1	3 × 2	6	0.003
S2	4 × 3	12	0.006
S3	6 × 4	24	0.013
S4	8 × 6	48	0.025
S5	12 × 8	96	0.05

**Table 4:** Comparison of measurement methods regarding utilization use cases.

Studied Problem	Precise Waveform Analysis	String Models Validation	Performance Analysis	Sound Synthesis Data	Instrument Timbre Analysis	Observing Vibration Polarities
**LDV**	Yes ^1^	Yes	No	Yes	No	Yes
**Microphone**	No	No	Yes	Yes	Yes ^1^	No
**Magnetic Pickups**	Yes	Yes	Yes	Yes	Yes	No

^1^ Most appropriate.

**Table 5:** Comparison of measurement methods in regards to limitations resulting from experimental scenarios.

Scenario	Nonferrous Strings	Light Strings	Instrument Mount Instability	Excitation Mechanism Noise	Noisy Environment	Electromagnetic Interference
**LDV**	Yes	No ^1^	No	Yes	Yes	Yes
**Microphone**	Yes	Yes	Yes	No	Yes	No
**Magnetic Pickups**	No	Yes	Yes	No	Yes	No ^2^

^1^ Due to requirement for optical target. ^2^ Single coil—no, Humbucker—conditionally yes.

**Table 6:** Comparison of measurement principles, strengths, limitations, and ideal use cases of different sensing methods for string vibration analysis.

Feature	Laser Doppler Vibrometer (LDV)	Microphone	Magnetic Pickups
**Principle**	Measures transverse string velocity directly via Doppler shift of reflected laser light.	Measures acoustic pressure variations generated by the instrument.	Measures string velocity from variations in magnetic flux induced by vibrating ferromagnetic strings.
**Strengths**	**High Precision**: Exceptional micro-temporal fidelity; resolves fine waveform details; **High SNR**: Highest signal-to-noise ratio in a controlled environment; **Robustness**: Unaffected by acoustic and electromagnetic interference.	**Perceptual Relevance**: Captures the holistic acoustic event as a human would hear it; **Calibrated**: Standardized measurement tool for acoustic analysis.	**Pragmatic Compromise**: Good balance of high-fidelity waveform capture, practicality, and low cost; **Non-Invasive**: Does not require altering the string; **Acoustic immunity**: Unaffected by ambient acoustic noise.
**Limits**	**Invasive**: Requires reflective sticker that adds mass, unacceptably altering the vibration of thin strings; **High Cost**: Significantly more expensive than other methods; **Practicality**: Highly sensitive to setup and movement, limiting its use to controlled laboratory settings.	**Low Precision**: Inadequate for resolving fine-scale waveform phenomena on the string itself; **Low SNR**: Low signal level means it is susceptible to noise from the excitation mechanism and ambient sound; **Indirect Measurement**: Captures the radiated sound, not the direct string motion, introducing medium-related filtering and delays.	**Inherent Filtering**: Filters signal with non-flat frequency response, poor performance over 5 kHz; **Interference**: Vulnerable to electromagnetic interference; **Non-universal**: Only functions with ferromagnetic strings; **Consumer product**: No measurement equipment, uncertain repeatability, and performance limits.
**Use Case**	High-precision physical modeling and validation in controlled lab settings, but only for systems where the required optical target does not significantly alter the object’s dynamics.	Psychoacoustic research, perceptual timbre analysis of the entire instrument, or studies where the radiated sound is the primary subject of interest.	Applied guitar research, performance analysis, and waveform-accurate modeling where target stability cannot be maintained or the invasiveness of an LDV target is unacceptable.

## Data Availability

The data presented in this study are available upon request from the authors.
